# Editorial: Mitochondria in Renal Health and Disease

**DOI:** 10.3389/fphys.2021.707175

**Published:** 2021-07-30

**Authors:** Egor Plotnikov, Giuliano Ciarimboli

**Affiliations:** ^1^Belozersky Institute of Physico-Chemical Biology, Lomonosov Moscow State University, Moscow, Russia; ^2^Experimental Nephrology, Medicine Clinic D, University of Münster, Munster, Germany

**Keywords:** acute kidney injury, chronic kidney disease, mitophagy, nephrotoxicity, lipotoxicity, antioxidants

The mitochondria is a unique cell hub that integrates many signaling pathways and determines the fate of the cell. Proper functioning of the mitochondria is especially important for the kidney, since the kidney is one of the most energy-demanding organs. Energy is required to sustain the intense transport processes, which constitute one of the most important renal function. In terms of the relative rate of oxygen consumption, kidney mitochondria are in second place after the heart (Boveris et al., 2006), ahead of the liver and brain. In addition, the kidney mitochondrial abundance is higher, compared to the brain and heart, second only to the liver (Schulz et al., 2015). While the production of energy and ATP is the key function of the mitochondria in the kidney, these organelles also regulate such processes as apoptosis, steroid synthesis, calcium and iron homeostasis, and a number of others that are discussed in the Topic.

It is known that the main substrate for energy production in the renal tubules are fatty acids, so in the Research Topic several papers discuss the effect of kidney mitochondria on lipid metabolism and the relationship of disorders in β-oxidation of fatty acids and the development of various kidney pathologies. Thus, in review by Lin and Duann, the recent advances in understanding of lipid metabolism role in the function of kidney mitochondria and the molecular mechanisms related to dyslipidemia during kidney disease progression are summarized. Authors discuss molecules targeting mitochondrial lipid metabolism and dysfunction, including pharmacological agents promoting mitochondrial fatty acid oxidation, mitochondrial biogenesis, and ATP synthesis, as well as mitochondrial antioxidants and cardiolipin stabilizers.

In the review by Console et al. the network of transporters and enzymes responsible for the mitochondrial fatty acid oxidation is considered in detail, focusing on derangements that underlie acute kidney injury (AKI). Restoring mitochondrial β-oxidation might reverse or attenuate renal failure. As the PGC-1α/PPARα axis governs transcriptional regulation of fatty acid oxidation, it was proposed as therapeutic target in AKI.

The complex interaction of mitochondria and lipotoxicity is discussed in detail in a review by Ge et al. They prove that there is a vicious cycle when stress-induced reactive oxygen species (ROS) production inhibits mitochondrial fatty acid β-oxidation and lipid utilization, which causes lipotoxicity and, in turn, increased ROS-generation. Thus, authors provided the evidence that both altered mitochondrial function and lipid metabolism may contribute to the pathogenesis of kidney diseases via lipid accumulation. Therefore, identification of therapeutic targets that break this vicious cycle may offer novel therapeutic interventions.

High energy-demand is characteristic of both the kidney and the muscles that is why mitochondrial dysfunction is a common event for pathologies such as chronic kidney failure and sarcopenia. Chronic kidney disease (CKD) is an illness that by itself exhibits sarcopenia symptoms. In the review by Takemura et al. the data indicating mitochondrial dysfunction in the skeletal muscle of patients with CKD are reported. Authors discuss therapeutic strategies with some factors related to the mitochondria in CKD that could provide renoprotection.

One of the main consumers of energy produced by the kidney mitochondria are ion pumps, which provide the transport of organic compounds and inorganic ions through the tubular cells. Membrane proteins such as organic anion transporters and the organic cation transporters are highly expressed on the proximal tubular cells and carry a wide range of solutes across the plasma membrane. In addition to the natural metabolites, these carriers are able to transport a number of xenobiotics, including drugs. As a result of accumulation of such nephrotoxic agents, a drug-induced AKI can develop. The mechanisms of this accumulation of drugs and their negative impact on the functioning of mitochondria are discussed in the review by Gai et al.

In addition to the excretion of drugs and xenobiotics, the kidneys maintain the homeostasis of various metal ions, whose amount can raise excessively because of some pathologies (hemochromatosis, hemoglobinuria) or consumption with food. Mitochondria are the main buffer and storage for ions such as iron, copper, manganese, and calcium, and on the other hand, it can suffer from their toxic effects. These cross-talk between mitochondria and metal ions are discussed in the review by Thévenod et al. The authors showed that the renal mitochondria have special transporters for iron in the outer mitochondrial membrane, which are also able to transport cadmium. The accumulation of these metals in the kidney mitochondria leads to its damage and the development of nephrotoxicity and AKI.

Calcium ions, which are also buffered by the mitochondria, are important for the development of urolithiasis. The association among mitochondrial dysfunction, intracellular or mitochondrial calcium concentration, and kidney stone pathogenesis are comprehensively discussed in the article by Chaiyarit and Thongboonkerd. They point that mitochondrial dysfunction alone is not sufficient to induce kidney stone formation, however, it is associated with oxidative stress and tissue inflammation in kidney.

The majority of renal pathologies are associated with the development of oxidative stress, in which mitochondria play a key role by producing ROS. Therefore, the antioxidant systems of the renal cells play a pivotal role in stress tolerance. The review by Kitada et al. examines the key functions of the mitochondrial antioxidant enzyme MnSOD, as well as the possibilities of correcting redox homeostasis with mitochondria-targeted antioxidants.

Finally, the importance of maintaining a pool of normally functioning mitochondria in kidney cells was demonstrated in the work of Zhu et al. The authors investigated the role of autophagy mediated by PINK/Parkin in the protection of kidney cells from ischemic damage. The protein augmenter of liver regeneration (ALR), which is ubiquitously expressed, stimulated the activation of mitophagy after ischemia/reperfusion, resulting in reduced mitochondrial dysfunction and production of ROS.

## Conclusion

Mitochondria are pivotal for maintaining the health and function of the metabolically active kidney by providing efficient energy support for maintenance of ions homeostasis and elimination of waste metabolites and environmental toxicants, including drugs. The articles collected in the Research Topic show that mitochondria are central element of several damaging cycles, such as lipotoxicity, accumulation of bivalent metal ions, and drug-induced AKI. In all these cases, protection of the renal mitochondria and effective mitochondrial quality control can be the factor that breaks the vicious circle and prevents the loss of kidney function.

## Author Contributions

Both authors listed have made a substantial, direct and intellectual contribution to the work, and approved it for publication.

## Conflict of Interest

The authors declare that the research was conducted in the absence of any commercial or financial relationships that could be construed as a potential conflict of interest.

## Publisher's Note

All claims expressed in this article are solely those of the authors and do not necessarily represent those of their affiliated organizations, or those of the publisher, the editors and the reviewers. Any product that may be evaluated in this article, or claim that may be made by its manufacturer, is not guaranteed or endorsed by the publisher.
